# Prognostic Value of Combined Hematological/Biochemical Indexes and Tumor Clinicopathologic Features in Colorectal Cancer Patients—A Pilot Single Center Study

**DOI:** 10.3390/cancers15061761

**Published:** 2023-03-14

**Authors:** Vladica Cuk, Aleksandar Karamarkovic, Jovan Juloski, Dragana Arbutina, Radosav Radulovic, Ljiljana Milic, Bojan Kovacevic, Silvio De Luka, Jelena Grahovac

**Affiliations:** 1 Zvezdara University Clinical Center, “Nikola Spasić” Surgical Clinic, Faculty of Medicine, University of Belgrade, Dimitrija Tucovica 161, 11000 Belgrade, Serbia; 2 Faculty of Medicine, University of Belgrade, Dr Subotica 1, 11000 Belgrade, Serbia; 3 Faculty of Dental Medicine, University of Belgrade, Dr Subotica 1, 11000 Belgrade, Serbia; 4 Department of Pathological Physiology, Faculty of Medicine, University of Belgrade, Dr Subotica 1, 11000 Belgrade, Serbia; 5 Department of Experimental Oncology, Institute for Oncology and Radiology of Serbia, Pasterova 14, 11000 Belgrade, Serbia

**Keywords:** biochemical indexes, colorectal cancer, prognostic value, tumor clinicopathologic features

## Abstract

**Simple Summary:**

Colorectal cancer (CRC) is a growing health burden in Serbia and worldwide. Surgical resection is the main modality for CRC treatment, and adjuvant treatment can further reduce the frequency of disease relapse and improve overall survival. Our study presents evidence that standard laboratory parameters, which do not present any additional cost for the health system, may provide additional information on the CRC patient outcome and lay the groundwork for a larger prospective examination. In our patient cohort, Clavien–Dindo classification of post-operative complications, modified Glasgow prognostic score, lymph node ratio, tumor deposits and peritumoral lymphocyte response were factors that were significantly associated with survival of operated patients.

**Abstract:**

Colorectal cancer (CRC) is a significant public health problem. There is increasing evidence that the host’s immune response and nutritional status play a role in the development and progression of cancer. The aim of our study was to examine the prognostic value of clinical markers/indexes of inflammation, nutritional and pathohistological status in relation to overall survival and disease free-survival in CRC. The total number of CRC patients included in the study was 111 and they underwent laboratory analyses within a week before surgery. Detailed pathohistological analysis and laboratory parameters were part of the standard hospital pre-operative procedure. Medical data were collected from archived hospital data. Data on the exact date of death were obtained by inspecting the death registers for the territory of the Republic of Serbia. All parameters were analyzed in relation to the overall survival and survival period without disease relapse. The follow-up median was 42 (24−48) months. The patients with the III, IV and V degrees of the Clavien–Dindo classification had 2.609 (HR: 2.609; 95% CI: 1.437−4.737; *p* = 0.002) times higher risk of death. The modified Glasgow prognostic score (mGPS) 2 and higher lymph node ratio carried a 2.188 (HR: 2.188; 95% CI: 1.413−3.387; *p* < 0.001) and 6.862 (HR: 6.862; 95% CI: 1.635−28.808; *p* = 0.009) times higher risk of death in the postoperative period, respectively; the risk was 3.089 times higher (HR: 3.089; 95% CI: 1.447−6.593; *p* = 0.004) in patients with verified tumor deposits. The patients with tumor deposits had 1.888 (HR: 1.888; 95% CI: 1024−3481; *p* = 0.042) and 3.049 (HR: 3.049; 95% CI: 1.206−7.706; *p* = 0.018) times higher risk of disease recurrence, respectively. The emphasized peritumoral lymphocyte response reduced the risk of recurrence by 61% (HR: 0.391; 95% CI: 0.196−0.780; *p* = 0.005). Standard perioperative laboratory and pathohistological parameters, which do not present any additional cost for the health system, may provide information on the CRC patient outcome and lay the groundwork for a larger prospective examination.

## 1. Introduction

Colorectal cancer (CRC) is a significant public health problem. Worldwide, in 2020, there were 1.9 million newly diagnosed CRC patients and 935,000 deaths from colorectal cancer [1]. Globally, every tenth newly ill and deceased patient with a malignant tumor had colorectal cancer. CRC is one of the most common cancers (after breast and lung cancer), with a 10% share of all malignancies, and the second leading cause of cancer death (after lung cancer) with a 9.4% share of all cancer-related deaths [1].

Surgical treatment is the main modality of CRC treatment [2]. Although it is generally known that tumor grade, degree of differentiation, histological (sub)type of tumor, tumor localization, number of positive lymph nodes and disease stage are good indicators of one-year, three-year and five-year survival rate, there are still controversies about prediction of colorectal cancer outcomes. There is no completely clear explanation for why patients with stage IIIa disease have better three-year and five-year survival rate than those in stage IIb (3-year survival: 91.4% vs. 80.2%; 5-year survival: 83.4% vs. 72.2%), which calls into question current treatment protocols [3]. Answers to these questions can be offered by recent research whose results go beyond generally accepted understanding of the biology of colorectal cancer [3,4]. There is increasing evidence that the host’s immune response and the nutritional status play a role in development and progression of cancer [5]. Taking into account the mechanisms of inflammation and the nutritional status of patients, great efforts are being made to find effective, easily accessible and cheap predictors of colorectal cancer prognosis in order to facilitate identification of critically ill patients, their postoperative monitoring and timely treatment. Several studies indicate that preoperative values of hematological/biochemical parameters (e.g., absolute values of lymphocytes, neutrophils, monocytes, platelets, values of serum albumin, C-reactive protein) and their mutual integration into indexes and scores are good indicators of the prognosis of colorectal cancer [6,7,8,9]. The LANR- specific ratio of absolute values of lymphocytes, neutrophils and albumin [6], PNI- prognostic nutritional index that integrates absolute values of lymphocytes and albumin [7], CAR- ratio of serum values of C-reactive protein and albumin [8], and mGPS- modified Glasgow prognostic score based on serum C-reactive protein and albumin [9], have been promising in predicting the patient outcome.

The majority of these findings stem from large cohorts of patients of the Asian population [6,7,8,10,11].

In the Republic of Serbia, the TNM stage of CRC and basic pathohistological findings, are taken as dominant indicators of prognosis and are often the only factors considered in deciding on adjuvant therapy. Preoperative nutritional status, standard hematological/biochemical parameters, and their integration into indexes in CRC patients have not been taken into account in CRC therapy decision making in Serbia to date. Our assumption was that the clinical laboratory results and the pathohistological status of tumor, which are generally available in every health institution around the world, present clinically “tangible” evidence of the subtle mechanisms of the immune system at the cellular and subcellular level that can help more precise prognosis for CRC patients and better-informed therapy decision making.

In this regard, the aim of our study was to examine the clinical markers/indexes of inflammation, nutritional and pathohistological status in relation to overall survival (OS) and disease-free survival (DFS) in Serbian colorectal cancer patients.

## 2. Materials and Methods

### 2.1. Patient Population

A database of 120 patients with diagnosed colorectal adenocarcinoma, confirmed by pathohistological findings, was retrospectively examined. All patients were operated on at the Surgical Clinic “Nikola Spasic” of Zvezdara University Medical Centre, in the period from January 2017 to December 2017. Excluding criteria were: (1) Presence of other malignancies treated in a period of less than 5 years preceding the time of surgery due to CRC; (2) Incomplete medical documentation; (3) Inadequate postoperative follow-up. Taking into account the exclusion criteria, after final processing a total of 111 patients met the study requirements. The stage of the disease was determined on the basis of the Eighth Edition of the American Joint Committee on Cancer (AJCC) Cancer Staging Manual [12]. The research was approved by the ethics board of the Zvezdara University Medical Centre in Belgrade, and all patients gave their written consent to participate in the research.

### 2.2. Patient Characteristics

Data on gender, age of patients, total length of hospitalization and length of hospitalization after surgical treatment were collected. The ASA (American Society of Anesthesiology) score was calculated for all patients, and postoperative complications were expressed through the Clavien–Dindo classification (C-D classification).

### 2.3. Preoperative Laboratory Measurements and Other Prognostic Scores

Data from standard biochemical and hematological analyses, performed in the hospital laboratory on Roche analyzers Cobas 6000 (c501 and e601) (Roche Diagnostics GmbH, Mannheim, Germany) and Sysmex XN-1000 (Sysmex Europe SE, Norderstedt, Germany) were issued by the hospital laboratory. In addition to the standard laboratory analyses, data on the preoperative values of sex hormones (Estradiol and Testosterone) and morning Cortisol as well as tumor markers (CEA, CA 19-9) were collected. All analyses were performed within a week before the operative treatment. 

Integrated hematological and biochemical parameters were calculated based on formulas used in previous studies: NLR (Neutrophile to Lymphocyte Ratio); MLR (Monocyte to Lymphocyte Ratio); PLR (Platelets to Lymphocyte Ratio); RLR (RBC to Lymphocyte Ratio); MPR (MPV to Platelets Ratio); CAR = CRP (mg/dL)/Serum albumin (g/dL); modified Glasgow Prognostic Score (mGPS): 0 (CRP ≤ 10, Alb ≥ 35), 1 (CRP ≤ 10, Alb < 35; CRP > 10, Alb ≥ 35), 2 (CRP > 10, Alb < 35); PNI (prognostic nutritional index) = Albumin value(g/L) + 5 × Lymphocyte (10^9^/L); LANR = Lymphocyte (10^9^/L) × Albumin (g/L)/Neutrophil (10^9^/L) [6,7,9,13].

### 2.4. Tumor Characteristics

Data on tumor localization, disease dissemination and type of surgery were obtained from the operative findings. Surgical preparations were analyzed by one pathologist and each pathohistological finding included: macroscopic description of the tumor, TNM classification, Astler–Coller classification, stage of the disease, tumor configuration, tumor size, macroscopic perforation of the tumor, macroscopic intactness of mesorectal fascia, histological type, histological grade, TIL (tumor-infiltrating lymphocytes), peritumoral lymphocyte (PTL) response of the tumor, mucinous component of the tumor, circumferential resection margin (in rectal cancer), lymphovascular, venous and perineural invasion, tumor deposits, total number of lymph nodes, number of positive lymph nodes, lymph node ratio, tumor budding. 

### 2.5. Follow-Up

At the Zvezdara University Medical Centre, postoperative follow-up was based on The National Guide of Good Clinical Practice for Colorectal Cancer issued by The Ministry of Health of the Republic of Serbia [14]. The national guide was modeled after the guide designed by Pfister et al. and represents a compromise between more and less intensive follow-up of patients after surgical treatment of CRC [15]. The overall patient survival (OS) was calculated as the date of diagnosis to the date of death from any cause. The disease-free patient survival (DFS) was defined as the time interval from cancer primary treatment until tumor recurrence or death from any cause. Tumor recurrence was defined as any clinically, biochemically or radiologically inferred relapse of the disease. Data on neoadjuvant, adjuvant chemo/radiation therapy and the disease recurrence for the included patients were collected from archived data at the Institute for Oncology and Radiology of Serbia in Belgrade and postoperative follow-up at the primary care health institution. In addition to in-hospital mortality, data on the exact date of death were obtained by inspecting the death registers for the territory of the Republic of Serbia. 

### 2.6. Statistical Analysis

In statistical analysis, continuous variables were expressed as mean ± standard deviation ($\bar{X}$ ± SD) or as median (interquartile range), while categorical variables were presented by number of cases (percentage). In order to assess the normality of the used data, the Kolmogorov–Smirnov test was used. The statistical computations for significance were two-tailed. The Mann–Whitney U test for continuous and the Chi-square test for categorical variables were used to assess differences between groups. All variables that showed a statistically significant correlation with disease survival and progression (*p* < 0.05) were analyzed by the Cox Hazard Ratio (Cox HR) model. The Cox HR model was used for univariate and multivariate regression analysis. Statistically significant differences between the analyzed variables (*p* < 0.05) in univariate analysis were included in multivariate regression analysis in order to assess good predictors of OS and DFS. Final outcomes were analyzed using the Log Rank test with the Kaplan–Meier survival curve. Statistical analysis was performed using IBM SPSS statistical software (SPSS for Windows, release 25.0, SPSS, Chicago, IL, USA).

## 3. Results

### 3.1. Patient Characteristics

Out of the 111 CRC patients that fulfilled the inclusion criteria, 56.8 % were male; predominantly the ASA (American Society of Anesthesiology) scores were 2 and 3. Among the 111 patients, 59.5% patients had a modified Glasgow Prognostic Score of 0. More than 85% of patients were older than 60 years. Clinical, biochemical and hematological laboratory characteristics of the patients are shown in Table 1. Differences in estradiol levels between male and female patients were statistically significant (*p* < 0.001) (median (interquartile range): 30.465 (18.35–60.015) vs. 18.35 (5–18.35), respectively), presumably due to the age of the majority of patients. Differences in estradiol levels between females younger than 60 and older than 60 years were statistically significant (*p* < 0.001).

Clinical characteristics of patients and pathohistological characteristics of tumors in relation to overall survival and disease progression after surgical treatment are shown in Table 2.

Out of the total number of operated patients, 34 (30.9%) patients had rectal cancer, while two (1.8%) had synchronous colon adenocarcinomas. Almost all patients, 106 (95.5%), underwent elective surgical treatment. Intrahospital mortality was observed in 6 (5.4%) patients. The median duration of postoperative treatment was 10 (8−12) days, and the total length of hospitalization was 13 (11−21) days. In our study, the follow-up median was 42 (24−48) months after surgery, while the median of survival without recurrence of the disease was 39 (10−45) months. During the mentioned follow-up period, 73 (65.8%) patients were still alive, while the recurrence of the disease was verified in 27 (27.6%) patients. 

### 3.2. Overall Patient Survival (OS)

Univariate and multivariate Cox regression analysis for overall survival period are shown in Table 3.

Taking into account the clinical characteristics of patients, with univariate and multivariate Cox regression analysis, we found that patients with III, IV and V degrees of the Clavien–Dindo classification had 2.609 (95% CI: 1.437−4.737; *p* = 0.002) times higher risk of death during a follow-up period of 42 (24−48) months. By analysis of gender, age, comorbidity and the received therapy, there was no statistically significant association with the total survival time (*p* > 0.05). 

By Cox regression univariate and multivariate analysis of hematological/biochemical characteristics, we found a statistically significant association with overall survival in patients with CRP > 10 mg/mL and serum albumin <35 g/L. Namely, patients with mGPS 2 had a 2.188 (95% CI: 1.413−3.387; *p* < 0.001) times higher risk of death during the period of our postoperative follow-up. In the univariate analysis, statistically significant association with the total survival time was also shown for the values of hemoglobin (*p* = 0.031), hematocrit (*p* = 0.030), CRP (*p* = 0.006), serum albumin (*p* = 0.001), CEA (*p* = 0.041), PLR (*p* = 0.045), CAR (*p* = 0.003) and PNI (*p* = 0.003), LANR (*p* = 0.035); while in the multivariate analysis none of them retained the statistical significance. The remaining analyzed hematological/biochemical parameters: NLR, MLR, RLR, MPR did not show a statistically significant association with the overall survival time. 

Regarding the pathohistological characteristics of colorectal cancer, univariate analysis showed a statistically significant association with the overall survival time in patients with stage III/IV disease (*p* = 0.009), LNR (*p* < 0.001), PTL response (*p* = 0.016), perineural invasion (*p* = 0.001), tumor deposits (*p* = 0.001) and tumor budding (*p* = 0.052). Taking into account all of these parameters, using multivariate Cox regression analysis, we found that patients with higher lymph node ratio had 6.862 (95% CI: 1.635−28.808; *p* = 0.009) times higher risk of death in the postoperative period; while this risk was 3.089 times higher (95% CI: 1.447−6.593; *p* = 0.004) in patients with verified tumor deposits.

**Figure 1:**
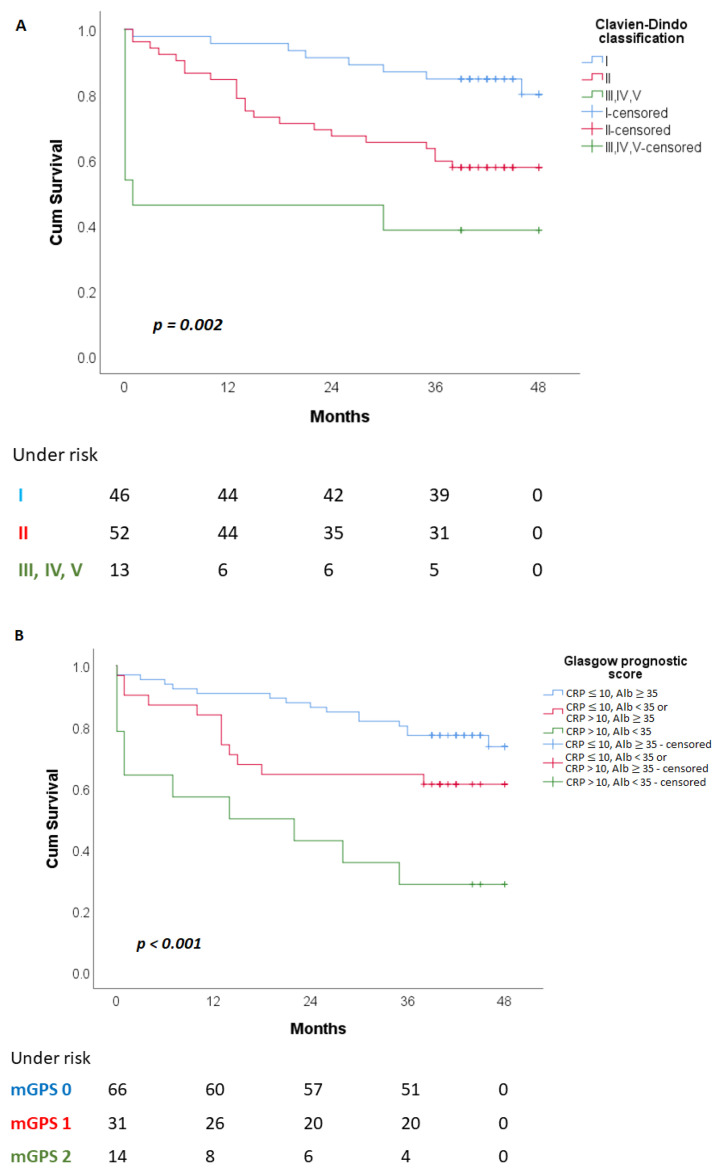

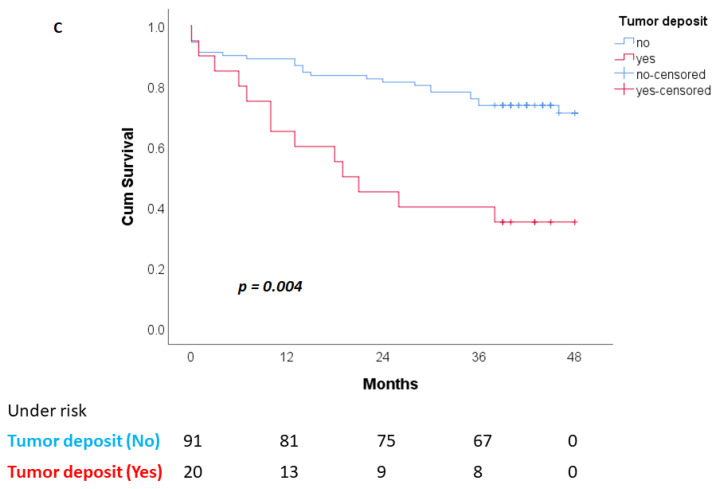
Kaplan–Meier curves for overall survival in patients grouped by: (**A**)—grades of complications by Clavien–Dindo classification; (**B**)—modified Glasgow prognostic score; (**C**)—presence of tumor deposits.

As expected, patients with grade III, IV and V complications of the Clavien–Dindo classification lived for a shorter period than patients with grade I and II complications (20.892 ± 6.318 vs. 43.688 ± 1.592 vs. 34,577 ± 2.415, respectively) (Figure 1A). Patients with mGPS 2 had significantly shorter average survival (21.45 ± 5.304 months) compared to patients with mGPS 0 (33.939 ± 3.365 months) and mGPS 1 (41.308 ± 1.681 months) (Figure 1B). Patients with tumor deposits had a significantly shorter survival (25.405 ± 4.162 months) compared to the patients without tumor deposits (39.240 ± 1.686 months) (Figure 1C).

### 3.3. Disease-Free Patient Survival (DFS)

Univariate and multivariate Cox regression analyses for DFS are shown in Table 4.

The age of the patients posed a risk for disease relapse in univariate regression analysis (HR: 0.961; 95% CI: 0.926−0.998; *p* = 0.041). Younger patients had a higher risk of disease recurrence, but this statistical significance was lost in multivariate regression analysis (*p* > 0.05).

None of the previously mentioned analyzed hematological/biochemical parameters and indexes had a significant statistical association with DFS.

Using univariate Cox regression analysis, a statistically significant association was found with survival time without relapse in stage III/IV disease (*p* = 0.002), LNR (*p* = 0.076), PTL response (*p* = 0.014), lymphovascular invasion (*p* = 0.069), perineural invasion (*p* = 0.035) and tumor deposits (*p* = 0.001). Multivariate regression analysis showed that patients with stage III/IV and tumor deposits had 1.888 (95% CI: 1024−3481; *p* = 0.042) and 3.049 (95% CI: 1.206−7.706; *p* = 0.018) times higher risk of disease recurrence, respectively. The emphasized peritumoral lymphocyte response reduced the risk of recurrence by 61% (HR: 0.391; 95% CI: 0.196−0.780; *p* = 0.005). 

**Figure 2:**
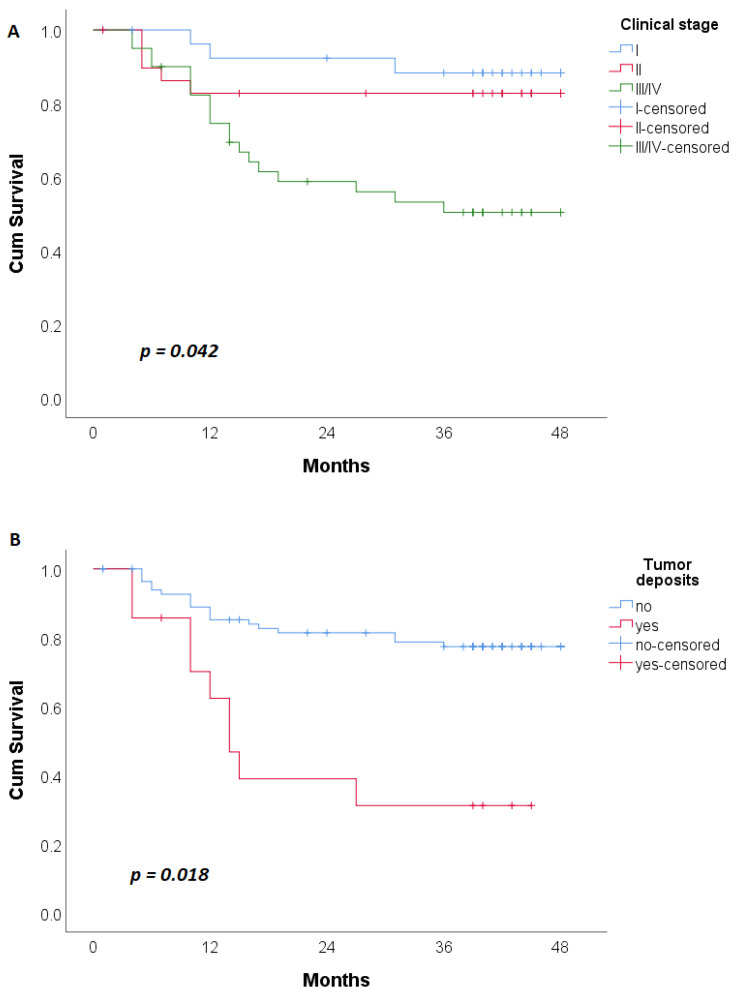

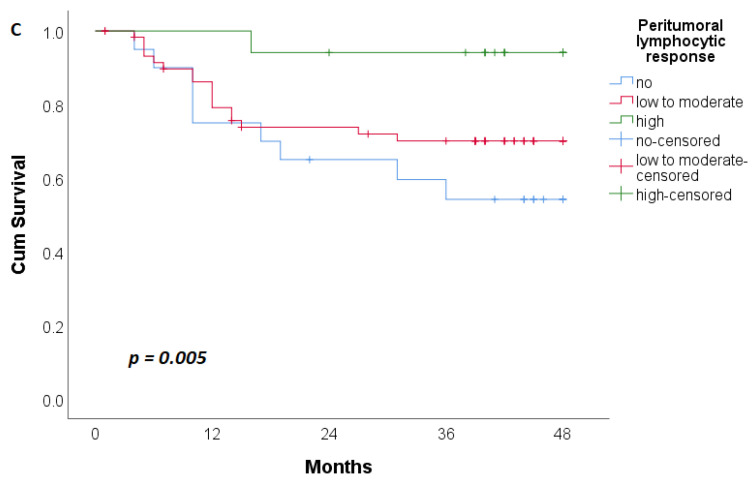
Kaplan–Meier disease-free survival curve in patients relative to: (**A**)—the TNM stage; (**B**)—presence of tumor deposits; (**C**)—peritumoral lymphocytic response.

Patients with clinical stage III/IV relapsed earlier than patients in stages I and II (31.506 ± 2.848 vs. 44.475 ± 2.010 vs. 40.828 ± 2.922 months, respectively) (Figure 2A). The same trend was observed in patients with tumor deposits (Figure 2B). Patients with high peritumoral lymphocyte response had longer disease-free survival time compared to patients without and to patients with mild to moderate peritumoral lymphocyte response (46.118 ± 1.826 vs. 33.429 ± 3.902 vs. 37.230 ± 2.244, respectively) (Figure 2C).

## 4. Discussion

In this study, the association of hematological/biochemical parameters, their indexes and pathohistological characteristics of the tumor with the CRC patient outcome were examined in a pilot single center cohort in Serbia. Colorectal cancer is a growing health burden in Serbia and worldwide. Although a Serbian national screening program for colorectal cancer in the population aged 50 to 74 was announced in 2013, to date the only screening method is colonoscopy and in practice no structured screening exists. Genetic testing for Lynch syndrome in Serbia was established in 2018. This has an impact on the characteristics of patient cohorts treated at the secondary and tertiary points of care. Nevertheless, examination of these groups provides real world insight into the clinical utility of prognostic parameters validated in settings with more advanced screening procedures. The most important findings of our research were that postoperative complications of grade III, IV and V according to Clavien–Dindo classification, mGPS 2, higher LNR and tumor deposits were statistically significantly associated with worse OS. Moreover, III/IV TNM disease stage and tumor deposits were statistically significantly associated with worse DFS, while the presence of peritumoral lymphocyte response was statistically significantly associated with better DFS. Our examined group consisted mainly of patients older than 60 years (85.57%) and hence we did not find significant influence of age on the overall survival. Patient sex was also not significantly associated with overall survival, unlike in larger cohort studies that show that women have substantial survival advantage [16]. We speculate that, given the age of the patients in our cohort, the protective effect of estrogen in women was lost, as was previously documented [17,18]. Indeed, the estradiol levels in female patients older than 60 years (*n* = 43) were significantly lower than in patients younger than 60 years (*n* = 5). Estrogen confers survival advantage in females through estrogen-regulated genes and cell signaling [19], and can control tumor growth by regulating the tumor immune microenvironment [20]. The level of estrogen receptor expression in most females 50 years of age and older is less than 10% [21] and out of the total number of women in our study, 95.83% were postmenopausal women over the age of 50. With regards to age, by univariate regression analysis we noticed that younger patients had a higher risk of disease recurrence during three years of follow-up, but this significance was not confirmed in the multivariate regression analysis.

Our results showed that postoperative complications based on the Clavien–Dindo classification were an independent risk factor in relation to total survival time, but not in disease-free survival time (DFS). Clavien–Dindo classification is a simple and feasible grading system of postoperative complications. Grades I and II represent surgical complications that can be solved by conservative treatment. Grade III complications require surgical, endoscopic or radiological intervention with or without anesthesia. Grade IV complications mean life-threatening complication (including CNS complications) requiring intermediate care/intensive care unit management. Grade V implies a complication that ends in death [22]. In our cohort, patients with grades III, IV and V of the Clavien–Dindo classification had a HR for total survival of 2.609 (95% CI: 1.437−4.737; *p* = 0.002), slightly higher than in the previously reported large observational studies [23,24].

Postoperative complications are partly due to the involvement of the immune system. Systemic inflammation is an indicator of poor prognosis in 21−41% of patients with colorectal cancer [25].

Many markers of systemic inflammation are based on the number, ratios or scores of circulating leukocytes or acute phase proteins, or serum albumins, such as NLR, LANR, MLR, CAR, mGPS [6,7,8,9]. Several studies have shown that NLR and LMR were good predictors of prognosis of overall survival, cancer-specific survival, and disease-free survival when considering cohorts of patients with rectal cancer or colon and rectal cancer together [26,27]. In our patient cohort, consisting of patients with both colon and rectal cancer, we did not find such associations for NRL and MRL. We also did not confirm the findings of Liang et al. [6], who were the first to report the LANR—the relationship between lymphocytes, serum albumin and neutrophils—as a good indicator of overall survival and relapse-free survival in resectable colorectal cancer. In our cohort, univariate analysis showed that LANR was associated with longer overall survival only (HR: 0.946; 95 CI: 0.898−0.996; *p* = 0.035), while multivariate analysis showed no statistical significance in terms of overall and disease-free survival.

The platelet to lymphocyte ratio (PLR) in our cohort had a significant statistical association with overall survival in univariate regression analysis only (HR: 1.002; 95% CI: 1.00−1.005; *p* = 0.045), while this statistical significance was lost in multivariate regression analysis. Ozawa et al. have shown that high values of PLR alone are an independent prognostic factor for disease-free survival and cancer-specific survival in stage II disease for CRC cancer [28]. We did not find that this variable was associated with survival when all the stages of CRC were analyzed together.

Systemic inflammation is a very important factor in cachexia related to malignancy. Cachexia not only reduces the quality of life of patients and the response to treatment, but is also an indirect cause of death in about 20% of patients who eventually die from cancer [29]. Nutritional status plays a significant role in the overall survival in colorectal cancer patients [9,30,31]. In our patient cohort, significant survival indicators related to the nutritional status of patients were CAR, PNI, and mGPS.

Modified Glasgow prognostic score (mGPS) is a prognostic score based on C-reactive protein (CRP) and serum albumin concentrations. The mGPS score ranges from 0 to 2. Patients with both an elevated CRP (>10 mg/L) and decreased albumin (<35 g/L) are assigned a score of 2, whereas those with either an elevated CRP or decreased albumin alone are assigned a score of 1. Patients with a normal CRP concentration and albumin level are assigned a score of 0 [9].

In our study, 12.6% of patients had mGPS 2. Univariate and multivariate regression analysis showed that mGPS 2 increased the risk of death more than 2-fold during 42 months of the postoperative follow-up. This was in line with the study by Proctor et al. [9] where mGPS was found to be an independent prognostic indicator in multi-cancer analysis for overall survival and tumor-specific survival, including colorectal cancer. A recent study with a significant focus on patient nutritional status and chronic inflammation, conducted by Son et al. [30] implied that mGPS and CAR taken together had better prognostic value than individually considered mGPS and CAR in patients with CRC. On the other hand, in the cohort with mismatch repair-deficient colorectal cancer there was no statistically significant difference between the High CAR Group vs. Low CAR Group, either in terms of overall survival or in terms of survival time without disease relapse [32]. The results of the study by Nagashima et al. indicated that the mGPS was a good predictor not only of 60-day mortality, but also of the overall survival of patients with late-stage cancer and malignant bowel obstruction [33].

In our patient cohort, univariate regression analysis indicated that the ratio of CRP and serum albumin (CAR) was associated with longer overall survival (HR: 1335; 95% CI: 1102−1617; *p* = 0.003), without statistical significance in multivariate regression analysis. The same trend was observed in PNI (HR: 0.924; 95% CI: 0.877−0.974; *p* = 0.003), with neither CAR nor PNI values being a risk factor for disease recurrence. In contrast to our results, the results of the study by Tamai et al. propose CAR as an independent indicator of OS and imply that CAR is a useful and promising prognostic marker in elderly patients undergoing curative surgery for CRC [34].

CRP, an inflammatory marker, is an acute-phase reactant synthesized by liver cells [35]. An elevated CRP level reflects the inflammatory response caused by tumor necrosis and is significantly higher in metastatic colorectal cancer liver disease [36]. CRP is mediated by many pro-inflammatory cytokines [37], which suppress the synthesis of albumin under inflammatory conditions [38]. In our patient cohort, the number of patients with metastatic liver disease of CRC (n = 9), which leads to the highest oscillations in the values of CRP and serum albumin, was smaller than in other studies [36], so the results related to CAR were slightly different.

In terms of pathohistological characteristics, univariate regression analysis of the TNM classification, lymph node ratio, peritumor lymphocyte response, perineural invasion, presence of tumor deposits and tumor budding showed statistical significance; while multivariate regression analysis showed that only higher lymph node ratio and presence of tumor deposits were strong indicators of a poor prognosis of the overall patient survival. Univariate analysis also showed that TNM stage, lymph node ratio, peritumor lymphocyte response, lymphovascular invasion, perineural invasion and tumor deposits were associated with the disease-free survival in our cohort of CRC patients. In multivariate regression analysis the TNM stage, presence of peritumoral lymphocyte response and absence of tumor deposits were the only parameters associated with longer disease-free survival. In contrast to the systemic inflammatory response, which is associated with a poor prognosis [6,7,8,9], verified intense immune cell infiltration in and around the tumor is often associated with better survival in colorectal cancer, regardless of disease stage or other prognostic parameters [39]. This is attributed to the ability of immune cells to recognize transformed malignant cells and limit tumor growth (immunosuppression hypothesis) [39]. Peritumoral lymphocyte response reduced the risk of disease relapse by 61% in our patient cohort, underlying the importance of the immune surveillance in cancer management [39]. The prognostic value of tumor deposits in our CRC patient cohort was in line with previous studies [40,41,42], confirming their importance as indicators of poor prognosis. While the disadvantages of our study were its single-center retrospective design, relatively small sample size and combination of colon and rectal cancer patients, as well as inclusion of patients with neoadjuvant chemoradiotherapy that would potentially alter their preoperative immune response, our results present a real world perspective on the utility of hematological/biochemical parameters and pathohistological characteristics of a tumor for prediction of survival of CRC patients in a middle-income Eastern European country. Insights that not all previously reported parameters hold prognostic value when examined in an age-skewed cohort lays the groundwork for examination of these specific parameters in a larger prospective study.

## 5. Conclusions

To the best of our knowledge, this is the first study of this type on the Serbian colorectal cancer patient population. It provides an important insight into the single center real-world scenario utility of the hematological/biochemical parameters, their indexes and pathohistological tumor characteristics as indicators of the prognosis in patients that were undergoing CRC surgery. These parameters—that are a part of any standard hospital pre-operative procedure and do not present any additional cost burden for the health system—may provide additional information on the patient outcome. We found that the Clavien–Dindo classification of post-operative complications, mGPS, lymph node ratio, tumor deposits and peritumoral lymphocyte response were factors worth taking into consideration when predicting survival of operated patients.

In combination with the reported genetic studies performed on the same population [43], our results may also be useful for future meta-analyses of CRC patient populations.

## Figures and Tables

**Table 1:** Clinical-pathological and laboratory characteristics of patients.

Characteristics of Patients	*n* (%)
Sex	
Male	63 (56.8)
Age (years) ^†^	67 (32−88)
<40 years	2 (1.8)
41–50 years	3 (2.7)
51–60 years	11 (9.9)
>60 years	95 (85.57)
ASA Score ^‡^	
1	18 (16.2)
2	50 (45.0)
3	43 (38.7)
4	0 (0)
5	0 (0)
Ten most common comorbidities	
Arterial hypertension (HTA)	81 (73.0)
Sideropenic anemia	30 (27.0)
Diabetes mellitus	29 (26.1)
Ischemic heart disease	21 (18.9)
Cardiac arrhythmias	15 (13.5)
Benign prostatic hyperplasia (BPH)	12 (10.8)
Chronic obstructive pulmonary disease	8 (7.2)
Hypothyroidism	7 (6.3)
Stroke	5 (4.5)
Renal failure	3 (1.8)
Hematological/biochemical values and Indexes §	
Leukocytes (109/L)	7 (5.70−8.4)
Erythrocytes (109/L)	4.51 (4.17−4.88)
Platelets (109/L)	293 (243−377)
Neutrophils (109/L)	4.72 (3.78−5.85)
Lymphocytes (109/L)	1.47 (1.14−1.94)
Monocytes (109/L)	0.35 (0.28−0.44)
Hemoglobin (g/dL)	12.10 (10.40−13.60)
Hematocrit (%)	37.70 (33.60−41.80)
RBC (RDW-CV) (%)	14.6 (13.3−17.4)
Serum albumin (g/L)	39 (35.00−42.00)
CRP (C-reactive protein) (mg/L)	4.80 (2.00−17.10)
CEA (ng/mL)	3.37 (2.02−9.11)
CA 19-9 (U/mL)	11.44 (6.28−28.46)
Cortisol 8 h (nmol/L)	425 (349.10−526.70)
Testosterone (nmol/L)	3.12 (0.32−10.64)
Estradiol (pmol/L)	21.18 (13.57−50.56)
Estadiol of males	30.465 (18.35–60.015)
Estadiol of females	18.35 (5–18.35)
Estadiol of females <60 years	30.75 (18.35–201.005)
Estadiol of females >60 years	18.35 (5–18.35)
NLR (Neutrophile to Lymphocyte Ratio)	3.09 (2.21−4.54)
MLR (Monocyte to Lymphocyte Ratio)	0.23 (0.18−0.32)
PLR (Platelets to Lymphocyte Ratio)	190.63 (141.60−276.14)
RLR (RBC to Lymphocyte Ratio)	10.23 (7.62−14.1)
MPR (MPV to Platelets Ratio)	0.03 (0.02−0.04)
CAR (CRP to Serum Albumin Ratio)	0.12 (0.05−0.45)
PNI (Prognostic Nutritive Index)	46.56 (42.30−50.85)
LANR (Lymphocyte, Serum Albumin, Neutrophile Ratio)	12.43 (7.78−17.76)
mGPS (modified Glasgow Prognostic Score) ^¶^:	
0	66 (59.5)
1	31 (27.9)
2	14 (12.6)

^†^ Data are shown as Median (min-max); ^‡^ ASA Score, American Society of Anesthesiology Score; § Data are shown as Median (25−75 percentiles); ^¶^ mGPS 0 (CRP ≤ 10, Alb ≥ 35), mGPS 1 (CRP ≤ 10, Alb < 35 or CRP > 10, Alb ≥ 35), mGPS 2 (CRP > 10, Alb < 35).

**Table 2:** Clinical and pathohistological characteristics in relation to overall survival and disease progression.

Parameters	Death		*p*	Relapse of Disease		*p*
	No *N* (%)	Yes *N* (%)		No *n* (%)	Yes *n* (%)	
Sex			0.300 *			0.892 *
Male	44 (60.3)	19 (50)		41 (57.7)	16 (59.3)	
Female	29 (39.7)	19 (50)		30 (42.3)	11 (40.7)	
Age (years) ^†^	66 (62−73)	68 (62−80)	0.117 **	67 (63−75)	63 (60−68)	0.045 **
ASA Score			0.248 *			0.032 *
1	11 (15.1)	7 (18.4)		9 (12.7)	8 (29.6)	
2	37 (50.7)	13 (34.2)		31 (43.7)	14 (51.9)	
3	25 (34.2)	18 (47.4)		31 (43.7)	5 (18.5)	
4	/	/		/	/	
5	/	/		/	/	
Diabetes mellitus	21 (28.8)	8 (21.1)	0.380 *	21 (29.6)	5 (18.5)	0.268 *
HTA	53 (72.6)	28 (73.7)	0.903 *	53 (74.6)	17 (63.0)	0.253 *
Leukocytes ^†^	6.9 (5.80−8.10)	7.65 (5.6−8.8)	0.218 **	7 (5.88−8.1)	7.7 (5.4−8.7)	0.811 **
Erythrocytes ^†^	4.55 (4.29−4.89)	4.3 (3.88−4.79)	0.059 **	4.48 (4.17−4.86)	4.57 (4.14−5.14)	0.375 **
Platelets ^†^	282 (228−373)	317 (258−389)	0.090 **	282 (228−375)	285 (230−392)	0.431 **
Neutrophils ^†^	4.59 (3.78−5.66)	5.18 (3.84−7.12)	0.196 **	4.68 (3.86−5.77)	5.05 (3.08−6.58)	0.880 **
Lymphocytes ^†^	1.54 (1.25−1.94)	1.33 (1.05−1.84)	0.254 **	1.51 (1.25−1.94)	1.56 (1−2)	0.990 **
Monocytes ^†^	0.34 (0.29−0.42)	0.35 (0.26−0.48)	0.751 **	0.36 (0.29−0.44)	0.32 (0.25−0.39)	0.183 **
NLR ^†^	2.89 (2.22−4.44)	3.73 (2.21−4.8)	0.160 **	2.9 (2.25−4.52)	2.9 (1.83−4.51)	0.833 **
MLR ^†^	0.22 (0.17−0.29)	0.25 (0.19−0.35)	0.234 **	0.22 (0.18−0.32)	0.22 (0.17−0.27)	0.278 **
PLR ^†^	188.3 (138.14−235.62)	211.64 (153.26−324.6)	0.056 **	187.5 (140.3−245.03)	193.88 (138.97−322.45)	0.559 **
MPR ^†^	0.03 (0.03−0.04)	0.03 (0.025−0.04)	0.229 **	0.03 (0.03−0.43)	0.03 (0.02−0.043)	0.933 **
RLR ^†^	9.375 (7.64−12.14)	12.3 (7.56−16.82)	0.093 **	9.71 (7.64−13.73)	9.3 (7.51−14.1)	0.759 **
CRP ^†^	3.5 (1.7−9.8)	10.3 (4−34.9)	0.001 **	4 (2−12)	6.1 (1.7−15.2)	0.605 **
Serum Albumin ^†^	40 (36−42)	37 (33−41)	0.005 **	40 (36−42)	41 (35−42)	1.000 **
Hemoglobin ^†^	12.4 (10.8−13.7)	11 (9.4−12.9)	0.037 **	12 (10−13.6)	12.3 (10.4−13.7)	0.535 **
Hematocrit ^†^	38.6 (35.2−41.9)	35.2 (31.9−41.7)	0.027 **	37.7 (33.6−41.7)	38.7 (32.6−42.9)	0.580 **
RBC (RDW-CV) ^†^	14.2 (13.2−16.7	14.9 (14−19.8)	0.041 **	14.7 (13.3−17.4)	14.4 (13.2−16.7)	0.404 **
CAR ^†^	0.08 (0.04−0.24)	0.257 (0.11−0.85)	0.001 **	0.09 (0.05−0.32)	0.16 (0.04−0.38)	0.611 **
PNI ^†^	47.3 (43.8−51.25)	54.1 (39.6−48.6)	0.013 **	46.7 (43.3−51.15)	47 (43.1−50.85)	0.849 **
LANR ^†^	13.27 (8.86−18.9)	9.85 (7.09−16.28)	0.043 **	13.27 (8.69−16.89)	12.43 (7.72−19)	0.852 **
CEA ^†^	2.99 (1.88−6.48)	5.2 (2.41−10.05)	0.030 **	2.76 (1.88−6.31)	3.59 (1.94−12.74)	0.361 **
CA 19−9 ^†^	10.41 (6.4−27.59)	14.33 (6.22−37.16)	0.230 **	10.92 (6.28−20.84)	12.01 (6.22−41.8)	0.324 **
mGPS			0.003 *			0.700 *
0	50 (68.5)	16 (42.1)		46 (64.8)	15 (55.6)	
1	19 (26)	12 (31.6)		19 (26.8)	9 (33.3)	
2	4 (5.5)	10 (26.3)		6 (8.5)	3 (11.1)	
Tumor Location			0.748 *			0.929 *
Right Colon	25 (34.7)	14 (37.8)		24 (34.3)	9 (33.3)	
Left Colon and Rectum	47 (65.3)	23 (62.2)		46 (65.7)	18 (66.7)	
Max Diameter of Tumor ^†^	40 (30−55)	40 (30−55)		40 (30−52)	40 (30−60)	0.582 **
TNM Stage			0.009 *			<0.001 *
I/II	46 (63)	14 (36.8)		50 (70.4)	8 (29.6)	
III/IV	27 (37)	24 (63.2)		21 (29.6)	19 (70.4)	
Number of Lymph Nodes ^†^	17 (12−21)	14 (12−17)	0.120 *	15 (12−21)	18 (13−21)	0.444 **
Positive Lymph Nodes ^†^	0 (0−1)	2 (0−5)	0.003 **	0 (0−1)	1 (0−5)	0.008 **
Lymphonodal Ratio (LNR)	0 (0−0.048)	0.113 (0−0.353)	0.001 **	0 (0−0.043)	0 (0−0.231)	0.008 **
Tumor Configuration			0.262 *			0.731 *
Exophytic	29 (39.7)	11 (28.9)		29 (40.8)	10 (37.0)	
Endophytic	44 (60.3)	27 (71.1)		42 (59.2)	17 (63.0)	
Tumor Gradus			0.148 *			0.193 *
G1	10 (13.7)	7 (18.4)		10 (14.1)	5 (18.5)	
G2	60 (82.2)	26 (68.4)		59 (83.1)	19 (70.4)	
G3	3 (4.1)	5 (13.2)		2 (2.8)	3 (11.1)	
TIL ^‡^			0.380 *			0.520 *
Without/Easy to Moderate	52 (71.2)	30 (78.9)		52 (73.2)	18 (66.7)	
Expressed	21 (28.8)	8 (21.1)		19 (26.8)	9 (33.3)	
PTL Response ^§^			0.019 *			0.029 *
Without	14 (19.2)	12 (31.6)		11 (15.5)	9 (33.3)	
Easy to Moderate	43 (58.9)	25 (65.8)		44 (62.0)	17 (63.0)	
Expressed	16 (21.9)	1 (2.6)		16 (22.5)	1 (3.7)	
Mucosal Component of The Tumor			0.401 *			0.292 *
Yes	23 (31.5)	15 (39.5)		21 (29.6)	11 (40.7)	
No	50 (68.5)	23 (60.5)		50 (70.4)	16 (59.3)	
Lymphovascular Invasion			0.205 *			0.038 *
Yes	45 (61.6)	28 (73.7)		39 (54.9)	21 (77.8)	
No	28 (38.4)	10 (26.3)		32 (45.1)	6 (22.2)	
Venous Invasion			0.015 *			0.020 *
Yes	0 (0)	3 (7.9)		0 (0)	2 (7.4)	
No	73 (100)	35 (92.1)		71 (100)	25 (92.6)	
Perineural Invasion			0.001 *			0.018 *
Yes	10 (13.7)	16 (42.1)		9 (12.7)	9 (33.3)	
No	63 (86.3)	22 (57.9)		62 (87.3)	18 (66.7)	
Tumor Deposits			0.001 *			0.001a *
Yes	7 (9.6)	13 (34.2)		5 (7.0)	9 (33.3)	
No	66 (90.4)	25 (65.8)		66 (93.0)	18 (66.7)	
Tumor Budding			0.037 *			0.838 *
Yes	55 (75.3)	34 (91.9)		56 (78.9)	21 (80.8)	
No	18 (24.7)	3 (8.1)		15 (21.1)	5 (19.2)	
Tumor Growth			0.113 *			0.752 *
Expansive	32 (44.4)	11 (28.9)		31 (44.3)	11 (40.7)	
Infiltrative	40 (55.6)	27 (71.1)		39 (55.7)	16 (59.3)	
Approach			0.092 *			0.926 *
Open	61 (83.6)	36 (94.7)		61 (85.9)	23 (85.2)	
Laparoscopic	12 (16.4)	2 (5.3)		10 (14.1)	4 (14.8)	
C-D Clasiffication ^¶^			0.003 *			0.598 *
I	38 (52.1)	8 (21.1)		32 (45.1)	11 (40.7)	
II	30 (41.1)	22 (57.9)		33 (46.5)	15 (55.6)	
III, IV, V	5 (6.8)	8 (21.1)		6 (8.5)	1 (3.7)	
Adjuvant CT ^#^			0.644 *			<0.001 *
Yes	20 (27.4)	12 (31.6)		13 (18.3)	15 (55.6)	
No	53 (72.6)	26 (68.4)		58 (81.7)	12 (44.4)	
Neoadjuvant CRT ^##^			0.619 *			0.744 *
Yes	4 (5.5)	3 (7.9)		4 (5.6)	2 (7.4)	
No	69 (94.5)	35 (92.1)		67 (94.4)	25 (92.6)	

* *p* values were calculated by the Chi-square test; ** *p* values were calculated by the Mann–Whitney test; ^†^ Data are shown as Median (25−75 percentiles); ^‡^ TIL, tumor infiltrating lymphocytes; ^§^ PTL response, peritumor lymphocytes response; ^¶^ C–D Classification, Clavien–Dindo Classification; ^#^ CT, Chemotherapy; ^##^ CRT, Chemoradiotherapy.

**Table 3:** Univariate and multivariate Cox regression analysis of overall survival in patients with colorectal cancer.

Parameters	Univariate		*p* Value	Multivariate		*p* Value
	HR	95% CI		HR	95% CI	
TNM Stage (III/IV) ^†^	2.430	1.254−4.711	0.009	/	/	/
LNR (Higher)	16.706	4.890−57.074	<0.001	6.862	1.635−28.808	0.009
PTL Reponse ^‡^ (Emphasized)	0.531	0.317−0.890	0.016	/	/	/
Perineural Invasion (Presence)	2.988	1.563−5.709	0.001	/	/	/
Tumor Deposits (Presence)	3.254	1.652−6.409	0.001	3.089	1.447−6.593	0.004
Tumor Budding (Presence)	3.233	0.992−10.540	0.052	/	/	/
C-D Classification Gradus III, IV, V	2.528	1.574−4.061	<0.001	2.609	1.437−4.737	0.002
Hemoglobin (g/dL)	0.844	0.723−0.985	0.031	/	/	/
Hematocrit (%)	0.938	0.885−0.994	0.030	/	/	/
CRP (mg/L)	1.009	1.003−1.016	0.006	/	/	/
Serum Albumin (g/L)	0.897	0.843−0.955	0.001	/	/	/
CEA (ng/mL)	1.010	1.000−1.019	0.041	/	/	/
PLR	1.002	1.000−1.005	0.045	/	/	/
LANR	0.946	0.898−0.996	0.035	/	/	/
CAR	1.335	1.102−1.617	0.003	/	/	/
PNI	0.924	0.877−0.974	0.003	/	/	/
mGPS 2	2.145	1.431−3.215	<0.001	2.188	1.413−3.387	<0.001

HR, Hazard ratio; CI, Confidence interval; ^†^ TNM Stage adopted binary classification (I/II vs. III/IV); ^‡^ PTL response, Peritumor lymphatic response.

**Table 4:** Univariate and multivariate Cox regression analysis of DFS in CRC patients.

Parameters	Univariate		*p* Value	Multivariate		*p* Value
	HR	95% CI		HR	95% CI	
Age	0.961	0.926−0.998	0.041	/	/	/
TNM Stage (III/IV) ^†^	2.486	1.390−4.445	0.002	1.888	1.024−3.481	0.042
LNR (Higher)	5.588	0.835−37.388	0.076	/	/	/
PTL Response (Emphasized)	0.465	0.252−0.858	0.014	0.391	0.196−0.780	0.005
Lymphovascular Invasion (Presence)	2.322	0.936−5.756	0.069	/	/	/
Perineural Invasion (Presence)	2.374	1.064−5.299	0.035	/	/	/
Tumor Deposits (Presence)	4.194	1.869−9.411	0.001	3.049	1.206−7.706	0.018

^†^ TNM Stage, adopted classification into three categories (I, II and III/IV).

## Data Availability

The data presented in this study are available on request from the corresponding author. The data are not publicly available due to ethical restrictions.
